# Facile synthesis of highly active fluorinated ultrathin graphitic carbon nitride for photocatalytic H_2_ evolution using a novel NaF etching strategy†

**DOI:** 10.1039/c8ra04691c

**Published:** 2018-07-30

**Authors:** Yanfei Liu, Junjie Wang, Chaochuang Yin, Huazhen Duan, Shifei Kang, Lifeng Cui

**Affiliations:** Department of Environmental Science and Engineering, University of Shanghai for Science and Technology Shanghai 200093 China sfkang@usst.edu.cn lifeng.cui@gmail.com; School of Chemical and Environmental Engineering, Shanghai Institute of Technology Shanghai 201418 China

## Abstract

Although graphitic carbon nitride (GCN) has been intensively studied in photocatalytic research, its performance is still hindered by its inherently low photo-absorption and inefficient charge separation. Herein, we report a simple NaF solution treating method to produce fluorinated and alkaline metal intercalated ultrathin GCN with abundant in-plane pores and exposed active edges, and therefore an enhanced number of actives sites. Compared to bulk GCN, NaF treated GCN has a larger specific surface area of 81.2 m^2^ g^−1^ and a relatively narrow band gap of 2.60 eV, which enables a 6-fold higher photocatalytic rate of hydrogen evolution.

## Abstract 2

Although graphitic carbon nitride (GCN) has been intensively studied in photocatalytic research, its performance is still hindered by its inherently low photo-absorption and inefficient charge separation.

## Graphical abstract

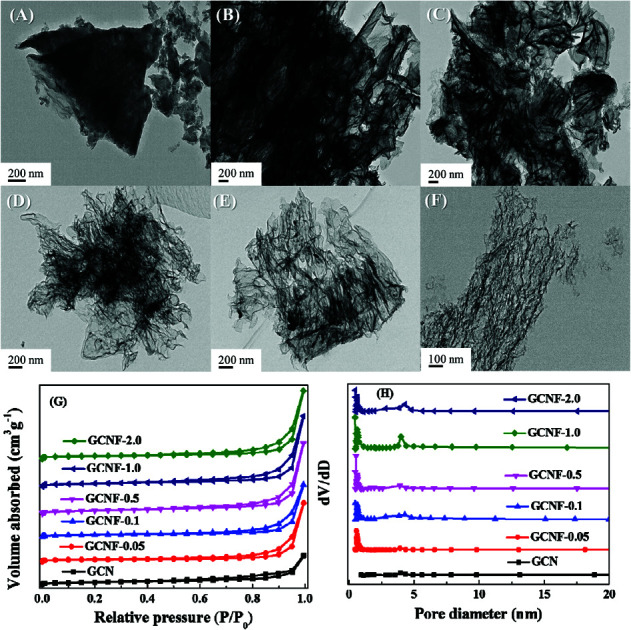

## Introduction

The worldwide energy shortage and environmental pollution have become serious threats to the sustainable development of human society. Many governments and scientists are trying to solve these problems through various green technologies.^1–3^ Among potential solutions, photocatalysis based on semiconducting materials is a prospective strategy that utilizes solar light and is also cost effective and environmentally friendly.^4,5^ The clean and renewable technology of photocatalysis has inestimable advantages, and only needs inexhaustible solar energy and suitable photocatalysts for various applications.^5^ Hydrogen fuel production has gained increasing attention as oil and other nonrenewable fuels become increasingly depleted and expensive, whereas hydrogen can be renewably produced using photocatalytic water splitting. As early as 1972, Fujishima and Honda co-authored the pioneering work of TiO_2_ electrodes for photo-electronic chemical water decomposition.^6^ However, existing photocatalysts are often inefficient because of the poor solar light absorption or the rapid recombination of photo-generated charge carriers. Since every photocatalytic reaction is mainly composed of three processes, photon absorption, generation and separation of electron–hole pairs, and catalytic surface reactions,^7^ any improvement to each of these three processes can result in enhanced photocatalytic activity.

Recently, non-conventional catalysts have also been extensively studied and some show excellent photocatalytic activity. For instance, in 2009 the pioneering work on GCN^8^ has drawn considerable attention because of GCN's unique electronic structure, high stability, and appropriate band gap. As a semiconductor with indirect band gap and a polymeric material made of only carbon and nitrogen, two rich elements in the earth, GCN is intrinsically a cheap and metal-free semiconducting polymer.^9,10^ Yet the photocatalytic performance of bulk GCN is unsatisfactory caused by the lack of active sites and the rapid recombination of photogenerated electrons and holes. Polymeric GCN can be easily and cheaply synthesized, and its properties can be adjusted without significantly changing its overall structure. Many strategies used to enhance the properties of various other materials were also applied on GCN. As an important modification method, fluorination has been adopted to improve the performances of graphite, activated carbons, carbon nanotubes, and BN nanotubes.^10,11^ On the other hand, the intercalation of alkaline metals (K^+^, Na^+^) into bulk GCN has been demonstrated to be able to increase the number of electron transportation channels.^12^ Therefore, simultaneous fluorination and alkaline metal intercalation is expected to greatly enhance the photocatalytic activity of GCN.

Herein, fluorinated ultrathin graphitic carbon nitride (GCNF) were synthesized by directly adding bulk GCN powders into NaF solutions of different concentrations for reaction and etching. The bulk GCN powder was synthesized by calcination of melamine in a muffle furnace. In a typical synthetic process, a designated amount of NaF (NaF mass *x* = 0.05, 0.1, 0.5, 1.0, 2.0 g) was dissolved in 100 mL of deionized water, then 0.5 g of bulk GCN powder was added into the NaF solution under stirring at room temperature for etching, exfoliation, fluorination, and Na^+^ intercalation. However, in the actual operation process, there is no ideal state Na^+^ and F^−^, which are present separately, and there is no suitable chemical reagent to replace. The NaF as a cheap and readily available raw material, which can meet the both functions at the same time. After centrifugation, washing and drying, the obtained samples were labeled as GCNF-*x*. In this simple NaF etching process, simultaneous fluorination and Na^+^ intercalation were achieved without the need of further high-temperature treatment, which is an inspiration for safe and clean production. The detailed experimental information of all test characterization in part 1 of the ESI.†

## Results and discussion

**Fig. 1:**
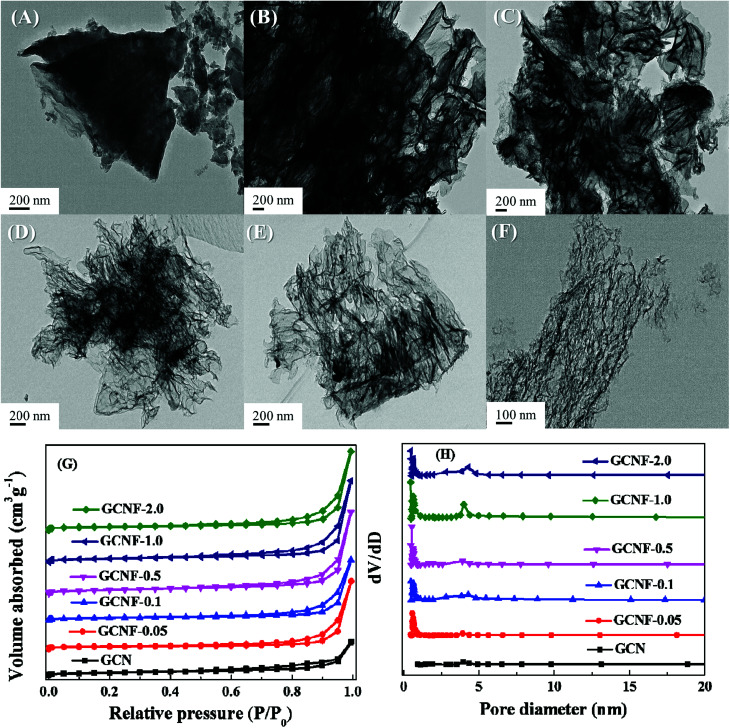
TEM images of (A) GCN, (B) GCNF-0.05, (C) GCNF-0.1, (D) GCNF-0.5, (E) GCNF-1.0, and (F) GCNF-2.0. (G) N_2_ adsorption–desorption isotherms and (H) Barrett–Joyner–Halenda (BJH) pore-size distributions of the six products.

The structure of GCNF was investigated by transmission electron microscopy (TEM), nitrogen adsorption–desorption measurements, X-ray diffraction (XRD), ultraviolet visible (UV-vis) diffuse reflectance spectrometry, X-ray photoelectron spectroscopy (XPS), photocurrent curves, electrons spin resonance (ESR), and Tafel curves. The morphology of the GCNF-based polymers was investigated with TEM. Clearly, the dosage of NaF greatly influenced the texture of the catalysts. When the dosage increased from 0.05 to 2.0 g, the compacted bulk GCN blocks were gradually corroded and exfoliated (Fig. 1A). The catalysts in Fig. 1B and C are irregular and thick blocks, which indicate slight erosion. However, the catalysts in Fig. 1D and E have nearly transparent two-dimensional sheet structures, which are more flexible and thinner.^12^ Finally, with the maximum dosage of NaF, the bulk GCN was extremely corroded and peeled into fragments (Fig. 1F). This special ultrathin structure endowed GCNF with more exposed edges. In addition, the obtained cross plane diffusion channels can further enhance the charge transport and separation of electrons–holes.^13^ In order to further prove the existence and distribution of in-plane pores in the product GCNF. We have provided the high-resolution transmission electron microscopy (HR-TEM) images of the GCNF-0.5 as shown in Fig. S1 of the ESI.† Furthermore, to determine the real thickness of g-C_3_N_4_ nanosheets. Atomic Force Microscope (AFM) was also performed and the results as shown in Fig. S2 of the ESI,† the ultrathin few-layer is predominant and the measured average thickness is 0.5 nm, which indicates the measured sheets are few-layer nanosheets. The nitrogen adsorption–desorption isotherms of GCN and GCNF-based polymers in Fig. 1G exhibit type-IV H3 hysteresis loops, and all the catalysts are in mesoporous structures.^14^ As expected, the GCNF-*x* polymers have larger specific surface areas (48.0, 59.3, 61.2, 81.2, 69.6 m^2^ g^−1^ respectively) than the bulk GCN (9.8 m^2^ g^−1^) (Table 1). The corresponding pore size distributions of the GCNF-*x* polymers show a wide peak from 0 to 20 nm (Fig. 1H), which can be attributed to the pores formed during the NaF corrosion.^15^ These data suggest NaF can introduce a porous structure with enlarged surface areas to promote photocatalytic mass transfer and reaction, which favor for enhancing the photocatalytic activity.

**Table 1:** S_BET_, pore volume and *E*_g_ (eV) of the six products

Product	Surface area (m^2^ g^−1^)	Pore volume (cm^3^ g^−1^)	*E*_g_ (eV)
GCN	9.8	0.09	2.70
GCNF-0.05	48.0	0.13	2.68
GCN-0.1	59.3	0.14	2.65
GCNF-0.5	61.2	0.17	2.63
GCNF-1.0	81.2	0.21	2.62
GCNF-2.0	69.6	0.18	2.60

**Fig. 2:**
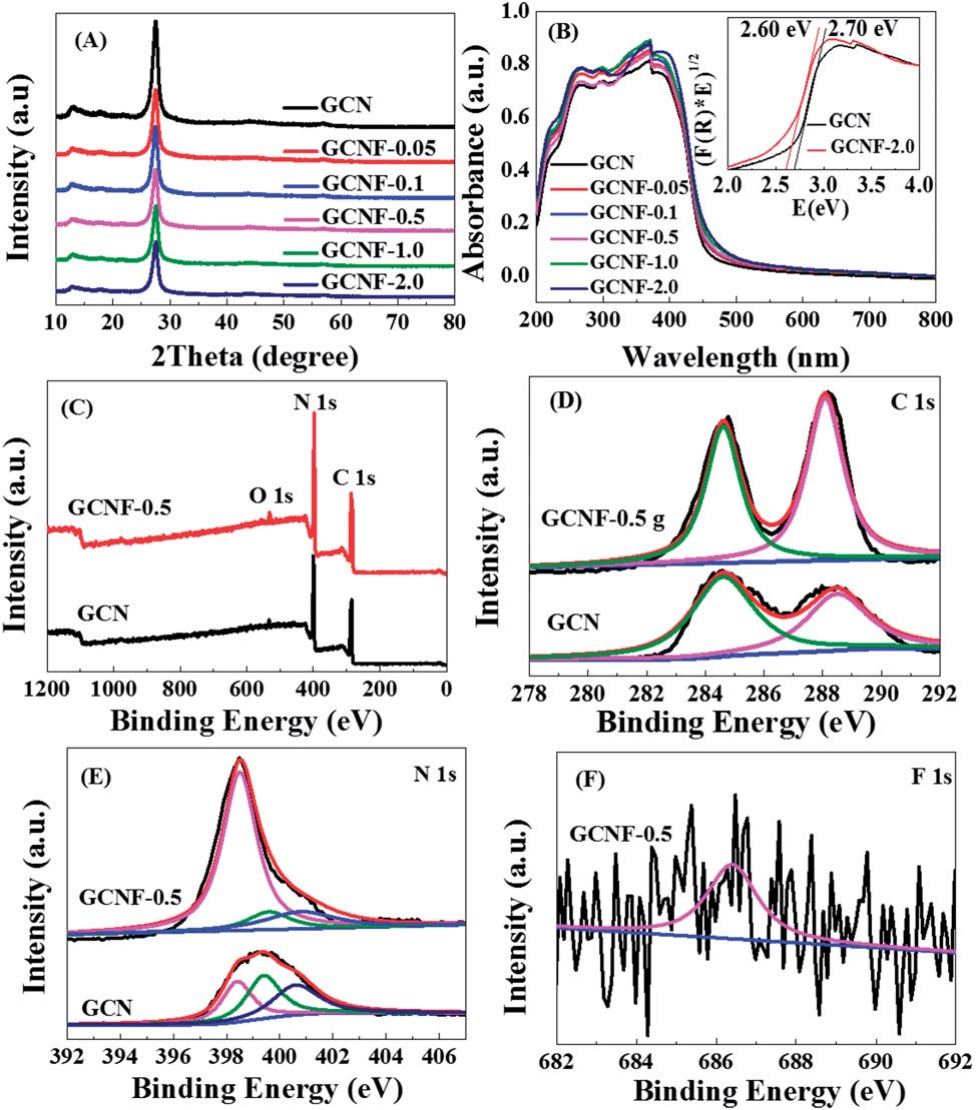
(A) XRD patterns and (B) UV-vis spectra of the six products, (B) and inset band gaps of GCN and GCNF-2.0. (C) XPS spectra of GCN and GCNF-0.5. High-resolution (D) C 1s, (E) N 1s of GCN and GCNF-0.5, and (F) F 1s peaks of GCNF-0.5.

The XRD patterns of GCN and GCNF-*x* (Fig. 2A) all reveal the graphite-like packing of two typical diffraction peaks at about 13.1° and 27.5°, which correspond to the (100) peak of the in-plane structural packing motif and the interlayer stacked of (002) peak for an aromatics fragment, respectively.^16^ Compared to the bulk GCN, the (002) peak is weakened gradually with the increased amount of NaF, indicating the interference of graphitic structure may be caused by the functional fluorine group. Thermogravimetric analysis (TGA) was conducted on a Perkin Elmer STA8000 thermal analyzer. There are two distinct stages of weightlessness of the GCN and GCNF-0.5 at 550 °C, first a major weight loss of the GCN about 70% because the crystal structure collapses, next a minor weight loss of the GCNF-0.5 about 8%, which indicating the GCNF-0.5 have improved thermal stability as shown in Fig. S3 of the ESI.† These data suggest the crystallinity and thermal stability of the GCNF have been obviously improved, which is beneficial for the photocatalytic performance and surface stability in aqueous solutions. The UV-vis spectra of GCNF-*x* show the optical band gap and semiconductor properties of GCNF materials have been changed slightly (Fig. 2B). The absorption of the spectrum extended to the visible light range and showed a remarkable blue shift. The fluorine incorporation led to the decrease of band gap from 2.70 eV of bulk GCN to 2.60 eV of GCNF-2.0, which was due to the extended π-delocalized system^17,18^ as confirmed by XPS and ESR. Therefore, compared with bulk GCN, the holey GCNF can absorb more visible light to produce more photogenerated charge carriers. The chemical states and compositions of the bulk GCN and GCNF-*x* were further studied by XPS. The XPS spectra (Fig. 2C) show the above products mainly compose of C and N elements. The trace O element can be attributed to the absorbed H_2_O or CO_2_ molecules on the surfaces of bulk GCN during the polymerization. The C 1s spectrum can be decomposed into two peaks centered on 288.5 and 284.6 eV (Fig. 2D), which are attributed to C=C and N–=C–N, respectively.^19,20^ The N 1s spectrum presents the characteristic peaks of three nitrogen statuses, including pyridinic-N (398.4 eV), triazine rings C–N–C (399.4 eV), and tertiary nitrogen N–(C)_3_ in heptazine unit (400.5 eV) (Fig. 2E).^21,22^ The XPS peak at 686.4 eV can be assigned to the fluorine attached to carbon and the fluorine concentration is of ∼2% (Fig. 2F), due to during the experiment, the fluorinated sample was cleaned with deionized water and collected by centrifugation, which resulted in the easily removal of without fluorinated ions. On the other hand, the peaks of non-metallic light elements are inherently weak in many physical and chemical tests, and the traces of F 1s displayed by existing XPS already are obvious, and which is similar to a previous literature.^5^ Although the Na^+^ ions was easily removed by deionized water during the experiment, we obtained sample about 0.59% of Na 1s by XPS characterization.

**Fig. 3:**
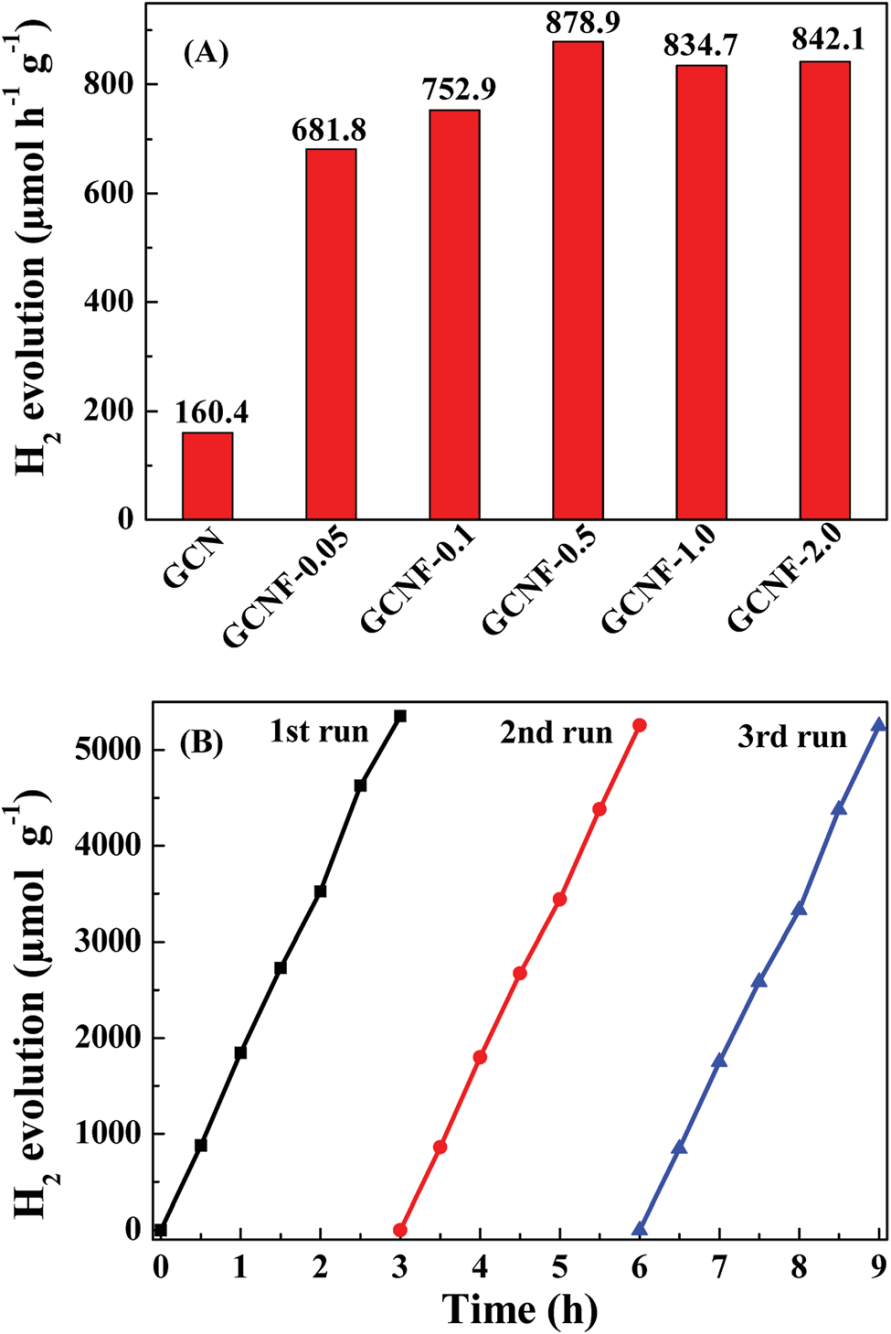
(A) H_2_ evolution rates of the six samples with 20 vol% triethanolamine solution, 3 wt% Pt as a co-catalyst and under visible light irradiation (*λ* > 420 nm). (B) Cycling test for photocatalytic H_2_ evolution activity of GCNF-0.5 under visible light irradiation (*λ* > 420 nm) (the closed gas circulation system was degassed again for 30 min; the light source was turned on after one cycle).

The photocatalytic performances of bulk GCN and GCNF-*x* (10 mg of a catalyst suspended in 20 mL of 20 vol% triethanolamine solution as a sacrificial agent) were evaluated by photocatalytic H_2_ release under visible light irradiation (a xenon 300 W lamp, *λ* > 420 nm) loaded with H_2_PtCl_6_ aqueous solution (3 wt% Pt) as co-catalyst. All the fluorinated catalysts show strengthened H_2_ production activity compared with GCN, and the H_2_ production rate maximized to 878.9 μmol h^−1^ g^−1^ at the NaF dosage of 0.5 g (Fig. 3A), which was about 6 times higher than the bulk GCN. Conventional bulk GCN contain many intermediates that are not fully polymerized, which have no photocatalytic activity. Corrosion can remove these incompletely polymerized intermediates thus improve photocatalytic performances. However, excessive corrosion also slightly removes the normally polymerized carbon nitride. When the dosage of NaF is 1.0 g and 2.0 g, the bulk GCN was extremely corroded and exfoliated into fragments as shown in (Fig. 1E and F). The photocatalytic activity of fluorinated ultrathin graphitic carbon nitride (GCNF) were closely related to their microtexture, so have a slight decrease of GCNF-1.0 and GCNF-2.0. Moreover, the H_2_ evolution rate of GCNF-0.5 was not significantly reduced after 3 runs in 9 h (Fig. 3B), which indicates the high stability of GCNF-0.5. The above results suggest the performance of photocatalytic H_2_ evolution is obviously improved due to the large specific surface area, short electron–hole transmission distance, and the increase of visible-light absorption.^23,24^ It is confirmed the innovative GCNF materials have the enhanced photocatalytic hydrogen production performance.

**Fig. 4:**
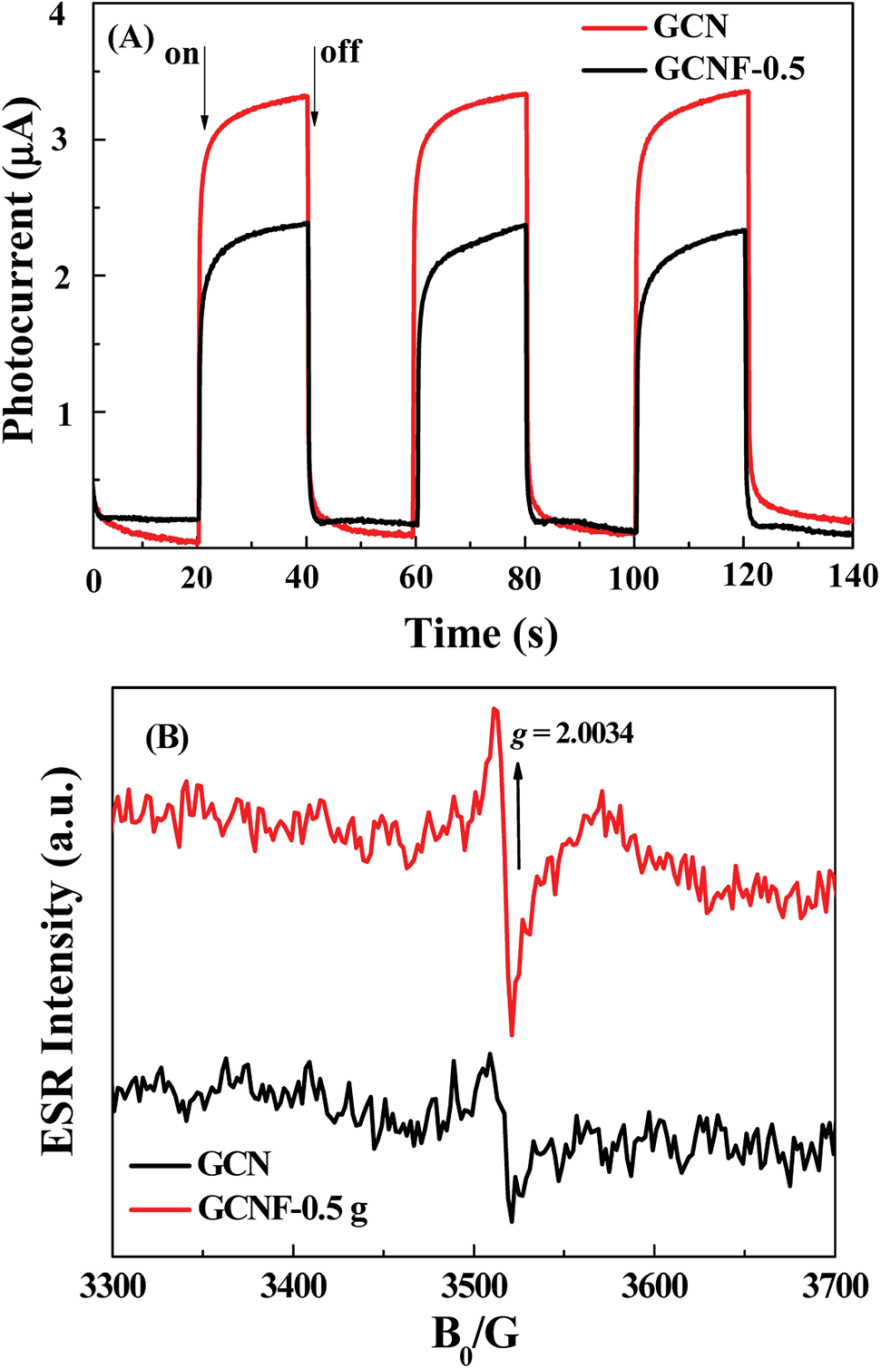
(A) Photocurrent–time dependence of GCN and GCNF-0.5 under visible-light irradiation. (B) ESR spectra of GCN and GCNF-0.5 at room temperature with a 300 W Xe lamp.

Electrochemical tests were carried out to further study the photo-induced charge transfer and separation behaviours. The photocurrent time curves of GCN and GCNF-0.5 are shown in Fig. 4A. Clearly, under the visible-light irradiation, the photocurrent response of GCNF-0.5 increased significantly compared with the bulk GCN, indicating GCNF-0.5 has less recombination and more effective separation of photoelectron–hole pairs.^25^ After the three repeated ON/OFF lighting cycles, the transient photocurrent between the photocatalysts was almost reversible and repeatable, which means high photoelectron chemical stability.^22,23^ The electronic structure was further characterized at room temperature through ESR powder. The images of GCN and GCNF-0.5 are different in composition (Fig. 4B), but the *g* values are similarly 2.0034 in the magnetic field of 3300–3700 G.^26,27^ The difference can be ascribed to different structures and electrochemical conditions between GCN and GCNF-0.5, and the increased ESR signals confirm the delocalization electrons are generated more due to the extended conjugation incorporated by aromatic rings. However, compared with the bulk GCN, the significantly stronger ESR spin intensity of GCNF-0.5 proves more enriched unpaired electrons, which contributes to the generation of active radical pairs of photocatalytic activity. These optical characteristics of GCNF-0.5 are very beneficial to the photocatalysis.^28,29^

**Fig. 5:**
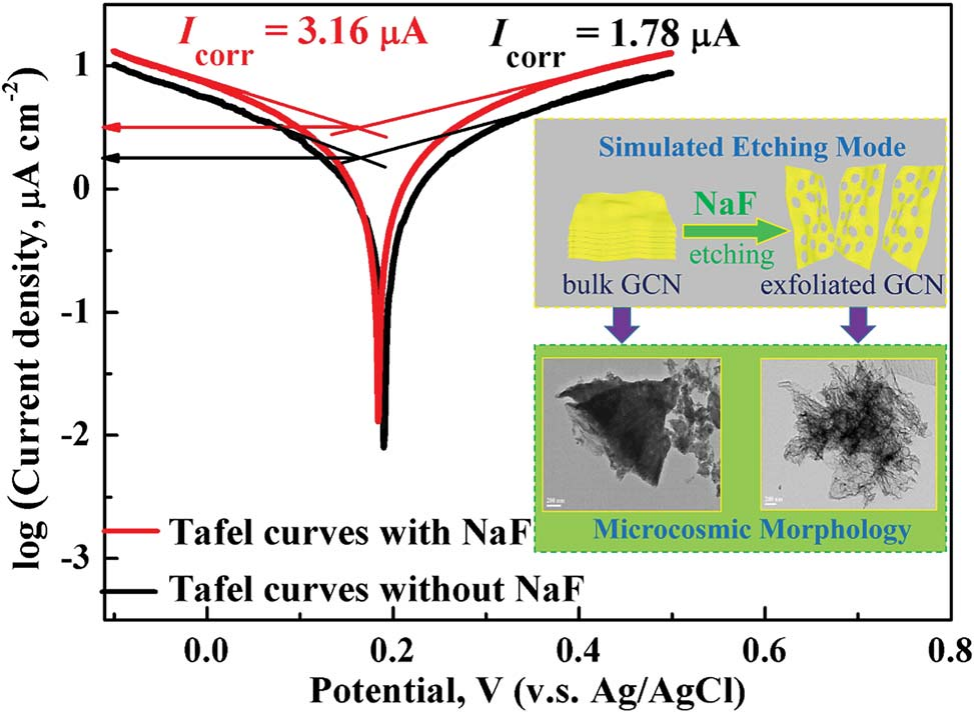
Tafel polarization curves of the bulk GCN with different solutions as the supporting electrolyte of 0.5 M Na_2_SO_4_ and 0.5 M Na_2_SO_4_ with 0.5 g NaF in 100 mL H_2_O at room temperature. Inset: schematic illustration of the corrosion and exfoliation of bulk GCN to form holey ultrathin GCN in NaF solution.

We proposed a schematic illustration about the formation of holey ultrathin GCN through corrosion and exfoliation of bulk GCN in NaF solutions (Fig. 5). Based on previously reported literature, the alkaline metal ions (K^+^ ion and Na^+^ ion) are inclined to be doped into the CN interlayers,^30–32^ which could enlarge the interlayer distance. Further, the F^−^ ion fluorination has been used to modify the properties of polymeric carbon nitride solids as reported.^5^ In this illustration, the general corrosiveness is the property of the reagent itself, the NaF not only has good corrosive properties, but also the intercalation effect of Na^+^ and the corrosion effect of F^−^ together effectively exfoliated the bulk GCN into ultrathin holey nanosheets.^33,34^ Moreover, to further verify the NaF corrosion mechanism, we measured the Tafel polarization curves using an electrochemical analyzer.^35^ The tangent slope of the anodic or cathodic branches of Tafel curve provides information about the corrosion current density. Compared with the bulk GCN in 0.5 M Na_2_SO_4_ solution in 100 mL of H_2_O, the bulk GCN in 0.5 M Na_2_SO_4_ with 0.5 g NaF in 100 mL H_2_O performed a larger slope in the anodic or cathodic branch and thus had a higher corrosion current density on the electrode (*I*_corr_ = 3.16 *vs.* 1.78 μA), which represent higher photocatalytic activity and lower charge transfer resistance at the reaction interface, which is consistent with photocurrent–time curves.^36,37^

## Conclusion

In conclusion, we present a new strategy to prepare fluorinated ultrathin graphitic carbon nitride based on NaF etching reaction. Fluorination not only can benefit the exfoliation, but also effectively regulates the electronic band gap to solve low photon absorption issue. As a result, GCNF has larger specific surface area, narrower band gap, more effective charge separation compared to the bulk GCN, and shows nearly 6 times higher visible-light-driven photocatalytic hydrogen evolution than GCN. Thus, the adjustment of morphology, internal electronic structure, and the band position proves effective in enhancing the photocatalytic activity of GCN. This study provides a novel strategy to adjust the physiochemical properties and electronic structures of a photocatalyst, and offers a simple and cheap approach to massively produce highly active fluorinated ultrathin graphitic carbon nitride for various catalytic applications.

## Conflicts of interest

There are no conflicts to declare.

## Supplementary Material

RA-008-C8RA04691C-s001
