# Supplementary material for: Facile synthesis of highly active fluorinated ultrathin graphitic carbon nitride for photocatalytic H2 evolution using a novel NaF etching strategy
Source: RSC Adv. 2018 Jul 30;8(48):27021–6. doi: 10.1039/c8ra04691c (PMC9083492; doi:10.1039/c8ra04691c)
Supplement: RA-008-C8RA04691C-s001 [file RA-008-C8RA04691C-s001.pdf]

Electronic Supplementary Material (ESI) for RSC Advances.
This journal is © The Royal Society of Chemistry 2018

Supplementary Information for

Facile synthesis of highly active fluorinated ultrathin graphitic carbon nitride for photocatalytic H_2 evolution using a novel NaF etching strategy

Yanfei Liu[a], Junjie Wang[a], Chaochuang Yin[a], Huazhen Duan[b], Shifei Kang[a]*, Lifeng Cui[a]*

a. Department of Environmental Science and Engineering, University of Shanghai for Science and Technology, Shanghai, 200093, China.

b. School of Chemical and Environmental Engineering, Shanghai Institute of Technology, Shanghai 201418, China.

Corresponding authors: Shifei Kang (E-mail: sfkang@usst.edu.cn); Lifeng Cui (E-mail: lifeng.cui@gmail.com)

Part 1 The detailed experimental information of all test characterization

The products were characterized on a JEM-2100F transmission electron microscope (TEM) at an accelerating voltage of 200 kV. Their nitrogen adsorption–desorption isotherms were measured on a Micromeritics Tristar 3000 analyzer at 77.4 K. X-ray diffraction (XRD) patterns were identified by using a Bruker Advanced D8 diffractometer (Germany) with Cu-Kα radiation and operated at 40 kV and 40 mA (scanning step: 0.02° s^{-1}) within 2θ of 10–80°. The diffuse reflectance spectra were analyzed in air with 200-800 nm wavelength range using a Shimadzu UV-2401 PC spectrophotometer (Agilent Technologies. USA) at room temperature, and $BaSO_4$ as reference material. X-ray photoelectron spectroscopy (XPS) spectrometer (Thermo Scientific, USA) were carried out on an ESCALAB 250 XPS instrument with monochromatic Al Kα. Photocurrents were measured with an electrochemical analyzer (Chi660e, Chen Hua Instruments, Shanghai, China). A standard three-electrode system adopts platinum wire, indium tin oxide (ITO) glass and Ag/AgCl (saturated KCl solution) as the counter electrode, working electrode and reference electrode, respectively. All the photocurrent measurements were performed under the illumination of a 300 W Xe lamp (λ > 420 nm), with 0.5 M Na_2SO_4 solution as the supporting electrolyte. Electrons spin resonance (ESR) signals were studied by using the 300 W Xe lamp as a visible-light source, and on the Bruker model A300 electron paramagnetic resonance spectrometer equipped with room temperature. Measuring Tafel polarization was carried out by an electrochemical analyzer (Chi660e, Chen Hua Instruments, Shanghai, China). A three-electrode cell arrangement was used for the electrochemical measurements, with a platinum wire, glassy carbon electrode and Ag/AgCl (saturated KCl solution) as the counter electrode, working electrode and reference electrode, respectively, with 0.5 M Na_2SO_4 solution as the supporting electrolyte. Among the working electrode was prepared by loading 20 μg of bulk GCN on a glassy carbon electrode of 5 mm in diameter (0.1 mg cm^{-2}). For the measurement 4 mg of sample was dispersed in 1 mL ethanol solution with 8 μL Nafion (5 %) and sonicated for 30 min. Finally, 5 μL of the dispersion was dropped onto the glassy carbon electrode for testing. Atomic Force Microscope (AFM) images were acquired in tapping mode on Japan Shimadzu SPM9700, using anhydrous ethanol as the dispersant, the supernatant was tested on a clean mica wafer. Thermogravimetric analysis (TGA) was conducted on a Perkin Elmer STA8000 thermal analyzer.

Part 2 High-resolution TEM image the GCNF-0.5.

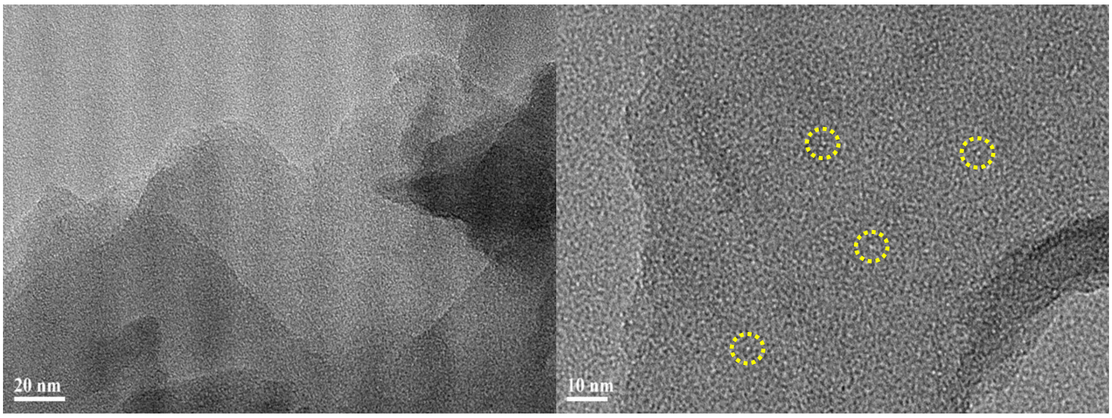

Figure S1. High-resolution TEM images the GCNF-0.5.

High-resolution TEM imagesw were characterized on a JEM-2100F transmission electron microscope (TEM) at an accelerating voltage of 200 kV. We have provided the high-resolution transmission electron microscopy images as shown in Figure S1, in order to further prove the existence and distribution of in-plane pores in the product GCNF.

Part 3 AFM images of the fluorinated graphitic carbon nitride GCNF-0.5

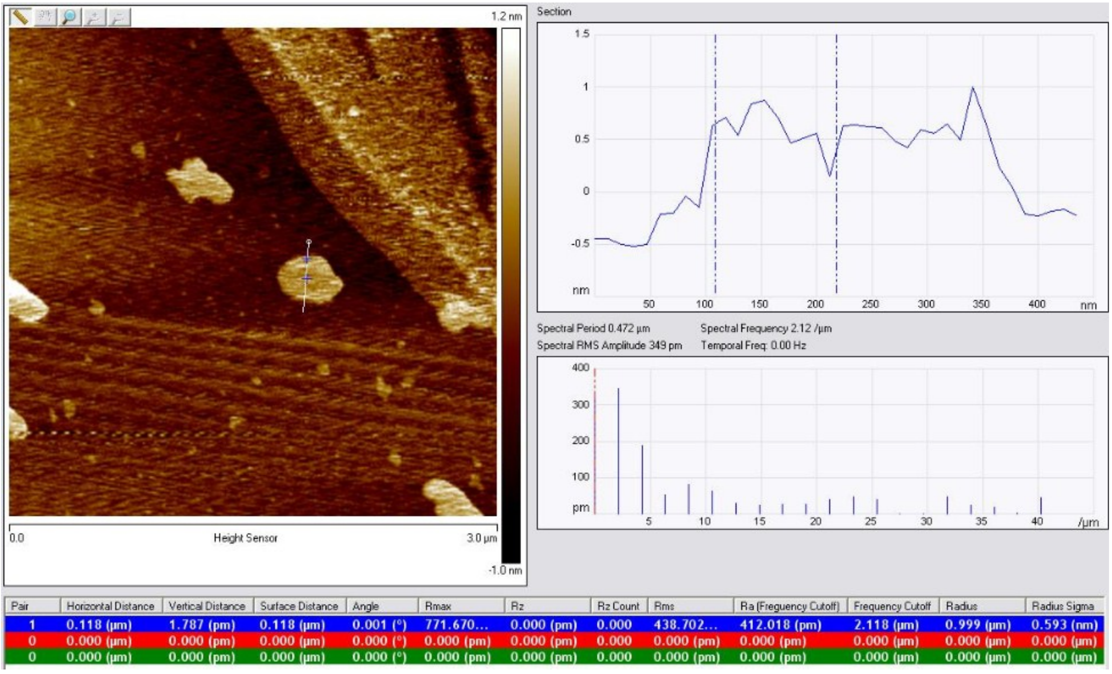

Figure S2. AFM image and corresponding height profile of the GCNF-0.5.

Atomic Force Microscope (AFM) images were acquired in tapping mode on Japan Shimadzu SPM9700. AFM results (Figure S2) clearly present a sheet-like object of ~ 200 nm in lateral size. Despite some overlaps observed in the images, the ultrathin

few-layer is predominant and the measured average thickness is 0.5 nm, which indicates the measured sheets are few-layer nanosheets, as the theoretical thickness of g-C_3N_4 is 0.33 nm [*Adv. Mater.* 2014, 26, 4438–4443.]. This finding directly proves g-C_3N_4 has been effectively delaminated into its few-layer sheets.

Part 4 The thermogravimetric analysis (TGA) curve of the GCN and GCNF-0.5.

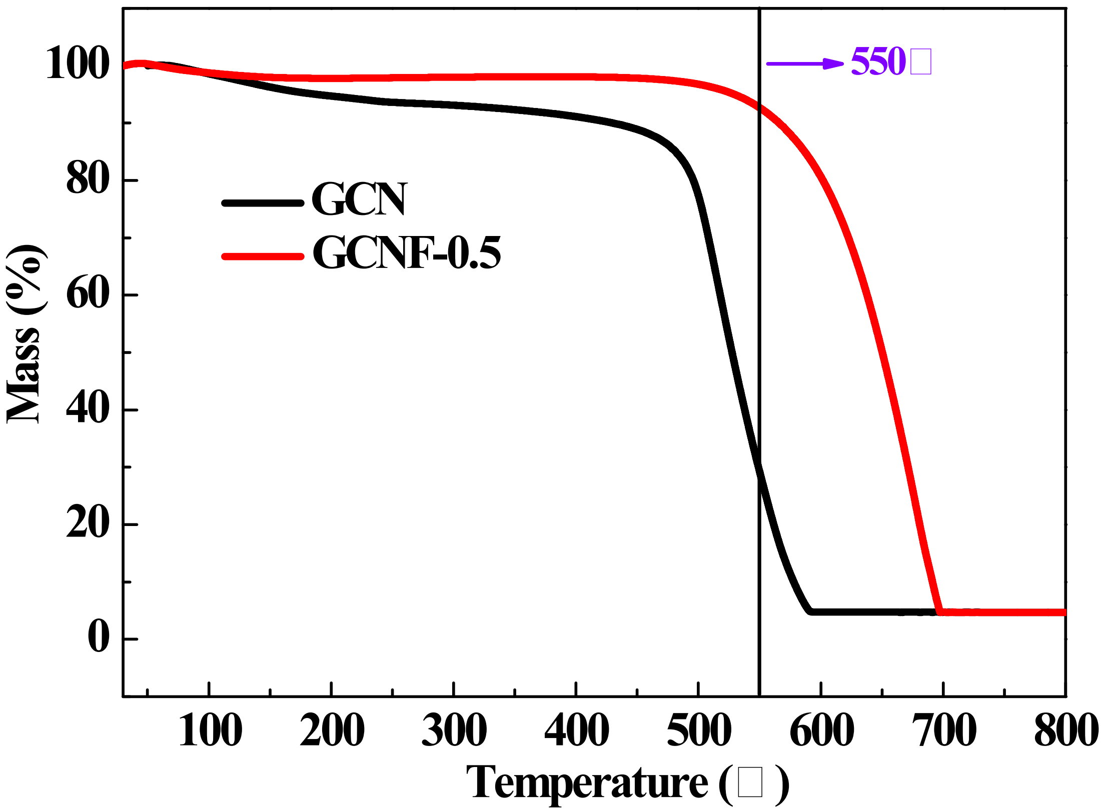

Figure S3. The thermogravimetric analysis (TGA) curve of the GCN and GCNF-0.5.

Thermogravimetric analysis (TGA) was conducted on a Perkin Elmer STA8000 thermal analyzer. In order to the direct evident to the GCNF have improved thermal stability, we have added the TG analysis as shown in Figure S3, which shows the thermogravimetric analysis (TGA) curves of the GCN and GCNF-0.5 in air at a heating rate of 10 °C min^{-1}. There are two distinct stages of weightlessness of the GCN and GCNF-0.5 at 550 °C, first a major weight loss of the GCN about 70% because the crystal structure collapses, next a minor weight loss of the GCNF-0.5 about 8%, which indicating the GCNF-0.5 have improved thermal stability.
